# Unilateral Diaphragmatic Paralysis: A Case Report of an Often Overlooked Diagnosis

**DOI:** 10.7759/cureus.64852

**Published:** 2024-07-18

**Authors:** Brianna Castellano, Riya Kumar, Minni Meka, Taraneh Honarparvar, Alejandro Biglione

**Affiliations:** 1 Medicine, Nova Southeastern University Dr. Kiran C. Patel College of Osteopathic Medicine, Fort Lauderdale, USA; 2 Internal Medicine, Wellington Regional Medical Center, Wellington, USA

**Keywords:** unilateral diaphragmatic paralysis, sob - shortness of breath, left flank pain, exertional dyspnea, diaphragm palsy, diaphragm dysfunction

## Abstract

Unilateral diaphragmatic paralysis, resulting from nerve or muscle injuries, is an uncommon phenomenon often missed due to its asymptomatic nature. This condition can lead to decreased pulmonary function, particularly in patients with underlying comorbidities or cardiopulmonary issues. Identification and understanding of the underlying cause of the paralysis are essential for effective management and improved patient outcomes. Here, we present a case of a 49-year-old male who presented with left flank pain and complained of dyspnea on exertion. Further workup and a sniff test confirmed the diagnosis of left hemidiaphragm paralysis.

## Introduction

Diaphragmatic paralysis occurs when the diaphragm loses its ability to contract and, subsequently, induces a negative intrathoracic pressure system during inhalation. This prevents the lungs from achieving proper expansion and ventilation. There are two types of diaphragmatic paralysis: bilateral and unilateral. Unilateral paralysis is the more common of the two and occurs when one hemidiaphragm, either the right or the left, loses the ability to move. This can occur from injury to the nerve supply or injury to the muscle itself. The presence of an elevated diaphragm is a common incidental radiologic finding. However, the diagnosis is relatively uncommon, and there is limited data on the incidence of true hemi-diaphragmatic paralysis in the general population. Diaphragmatic palsy can be due to various causes; however, injury or trauma to the diaphragm or phrenic nerve during surgery is the most common cause [1]. A less common cause is a result of penetrating neck trauma [2]. Diaphragmatic paralysis has the ability to cause a decline in pulmonary function, especially in at-risk patients with poor cardiopulmonary function, with a subsequent increased risk of morbidity and mortality [1]. Patients with unilateral diaphragmatic paralysis may also have limited ability to exercise, with an increased activation of larger accessory muscles [3]. Many patients present with little to no symptoms, thereby making it an often overlooked and neglected condition. Although patients can present asymptomatically, it is crucial to consider the complications in patients who present symptomatically or with co-morbidities. We present the case of a 49-year-old male who presented to our hospital due to left flank pain and dyspnea on exertion. The patient was found to have paralysis of the left hemidiaphragm.

## Case presentation

A 49-year-old male patient presented to the hospital with left flank pain and exertional shortness of breath. The patient denied any cough or chest pain upon initial presentation. He has a past medical history of recurrent urinary tract infections and pyelonephritis secondary to congenital ureteral stenosis that required the creation of an ileal conduit for urinary diversion. The patient presented with a temperature of 98.8°F, a pulse rate of 105 beats/minute, blood pressure (BP) of 108/69 mmHg, a respiratory rate (RR) of 18 breaths/minute, oxygen saturation of 99% on room air, and a body mass index (BMI) of 32.89. On physical examination, the patient presented with a soft, nondistended abdomen and left costovertebral (CVA) tenderness. The patient’s neck was supple with no jugular venous distention, heart sounds were regular in rate and rhythm with no murmurs, and the lungs were clear to auscultation bilaterally with equal breath sounds. No leg edema or lesions were noted.

**Figure 1:**
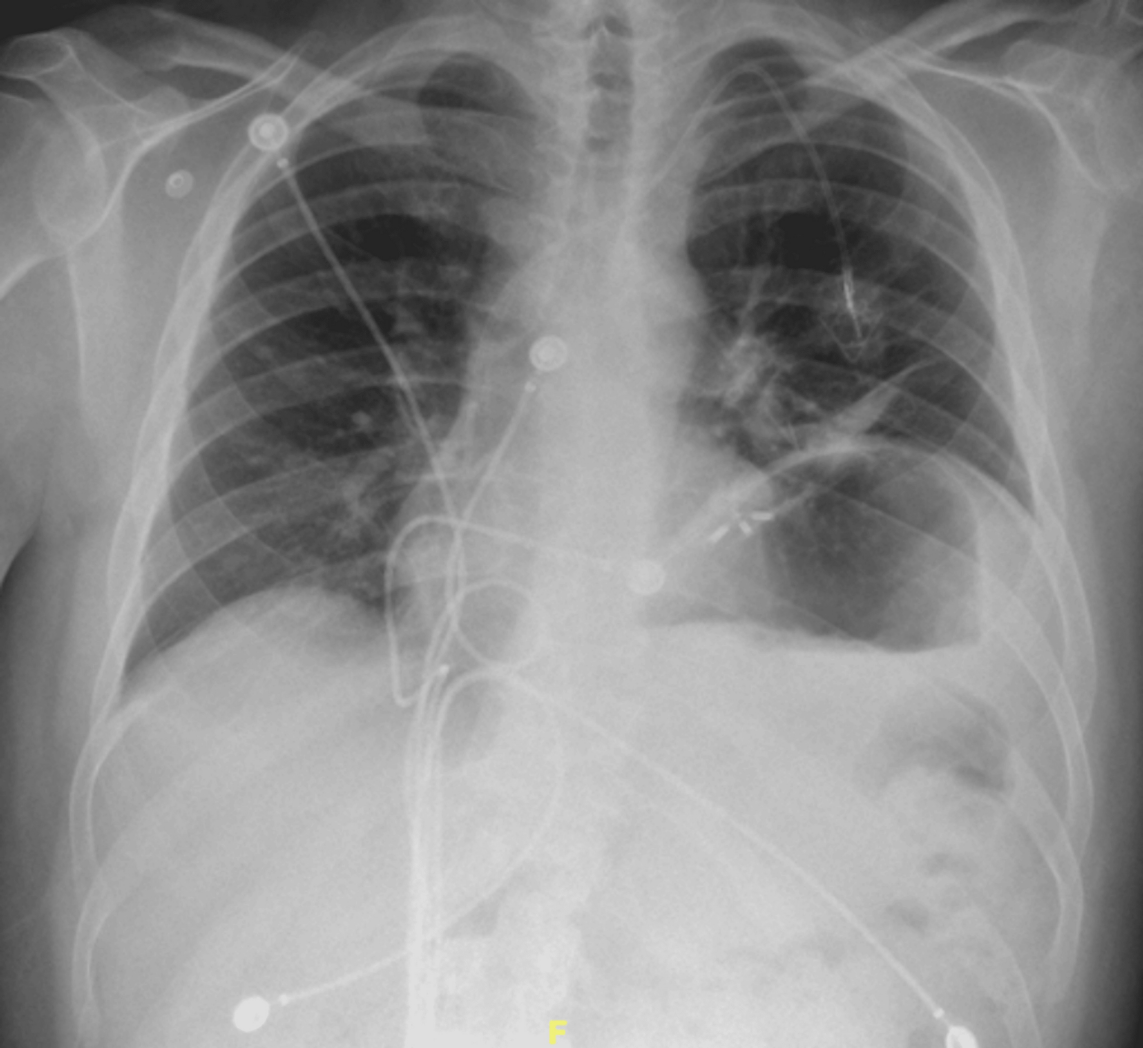
Chest radiograph reveals subsegmental atelectasis in the left lower lung zone and elevation of the left hemidiaphragm.

**Figure 2:**
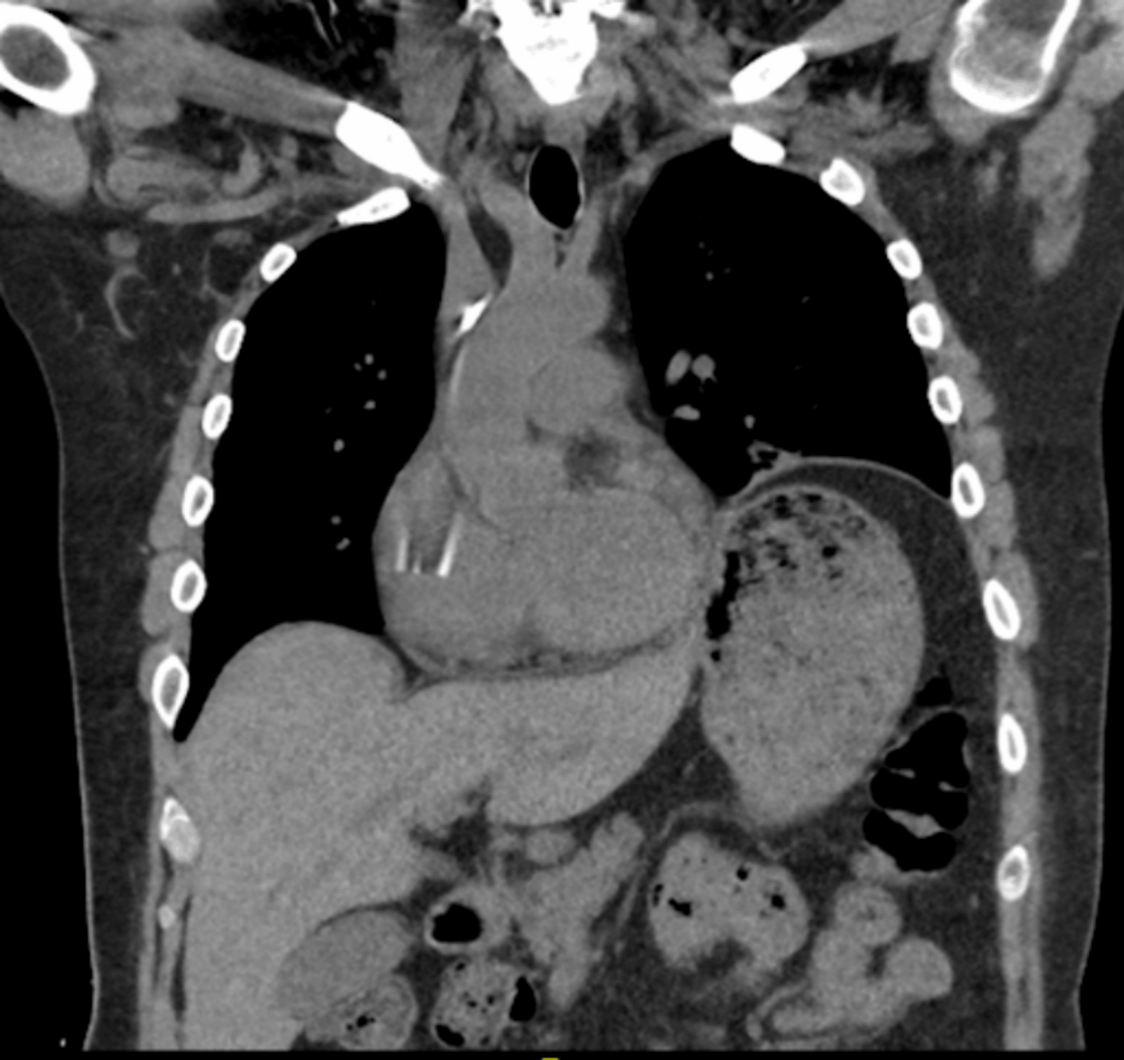
Computed tomography of the thorax reveals asymmetric elevation of the left hemidiaphragm and left basilar atelectasis or scarring.

**Figure 3:**
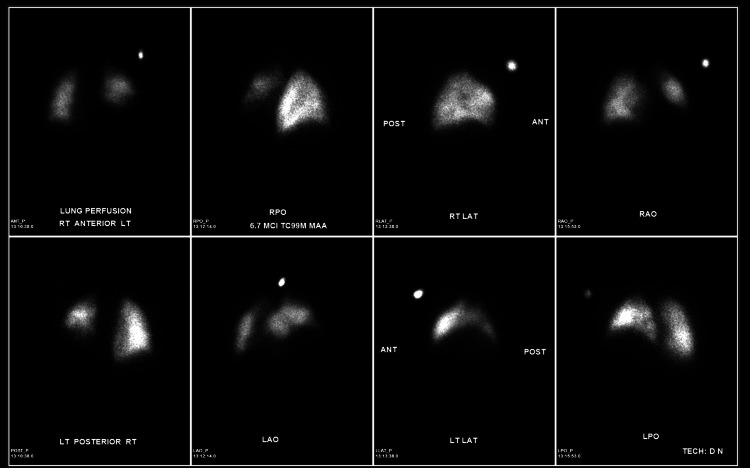
Limited ventilation and perfusion in the left lower lung zone, consistent with significant elevation of the left hemidiaphragm. Otherwise, no evidence of ventilation-perfusion mismatch.

**Figure 4:**
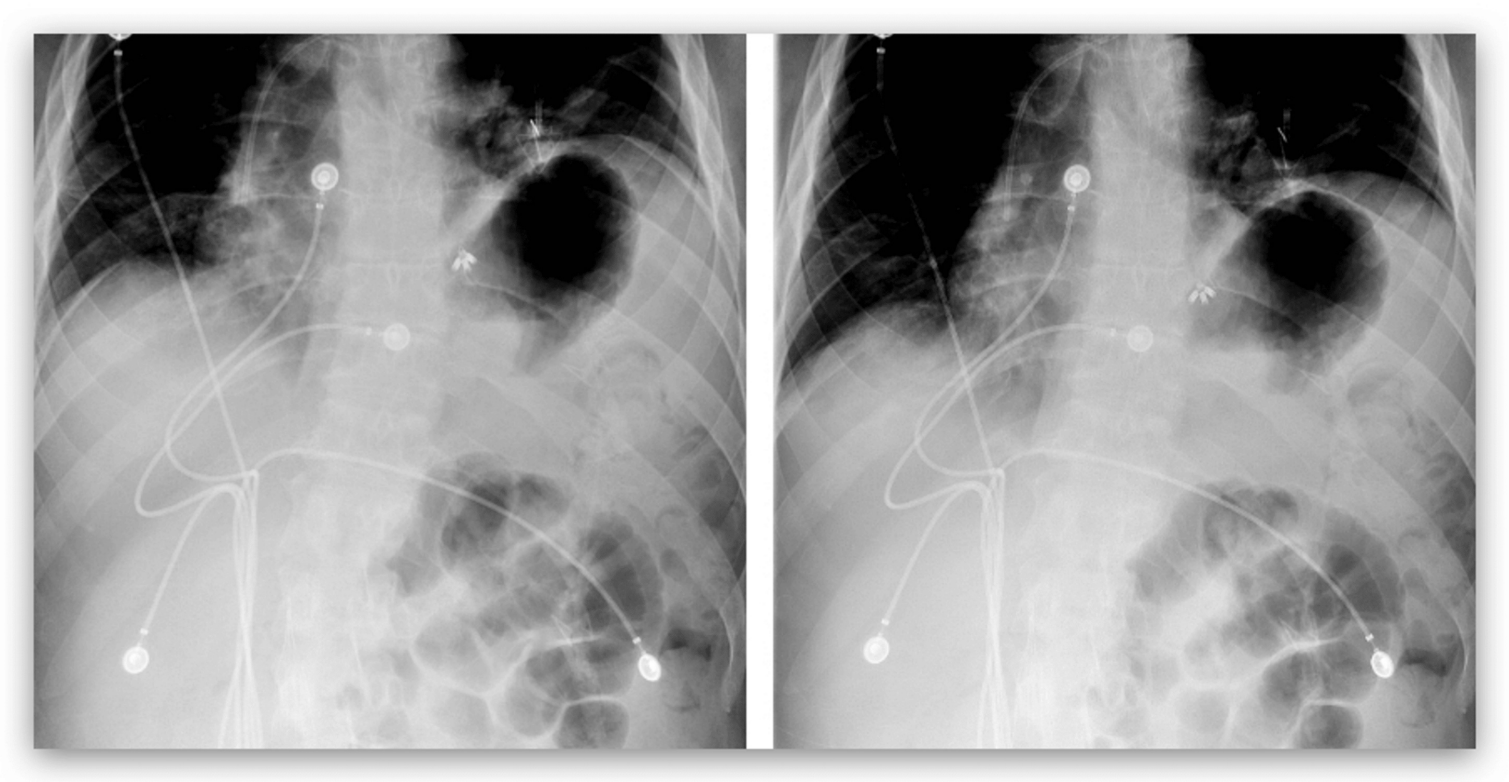
Sniff test reveals decreased left diaphragmatic excursion as compared to the right during inspiration (left image). Sniff test during expiration (right image).

The patient’s laboratory results demonstrated a glucose level elevated at 223 mg/dL, a creatinine level elevated at 1.88 mg/dL, a d-dimer level elevated at 2.75 mg/L, electrolytes, and liver enzymes within normal range, and BNP within normal range. The patient’s troponins were 4.4 ng/mL, and the electrocardiogram (EKG) was normal. Urinalysis demonstrated leukocyturia and bacteriuria, and the urine culture showed growth of *Enterococcus faecium*. A chest radiograph (CXR) was performed and showed subsegmental atelectasis in the left lower lung and elevation of the left hemidiaphragm (Figure 1). A computed tomography (CT) of the chest was then performed for further evaluation. It demonstrated asymmetric elevation of the left hemidiaphragm and left basilar atelectasis (Figure 2). For evaluation of suspected pulmonary embolism, nuclear medicine pulmonary perfusion with ventilation was chosen for imaging due to the patient’s elevated d-dimer and creatinine levels. Imaging revealed limited ventilation and perfusion in the left lower lung, consistent with significant elevation of the left hemidiaphragm (Figure 3). Diaphragm fluoroscopy (sniff test) was then performed to confirm the diagnosis of hemidiaphragmatic paralysis. The test demonstrated decreased left diaphragmatic excursion as compared to the right during inspiration (Figure 4), confirming clinical suspicion. The patient was managed with antibiotics for the urinary tract infection.

## Discussion

Unilateral diaphragmatic paralysis is an uncommon finding with limited data. The diaphragm is a crucial contributor to inspiration; therefore, dysfunction can be harmful. In patients with hemidiaphragm paralysis, during the inspiratory phase, the non-paralyzed diaphragm descends to uptake air through the trachea. Simultaneously, on the paralyzed side, air is drawn across the chest into the unaffected lung, causing paradoxical (upward) movement of the affected hemidiaphragm. This upward motion leads to basal atelectasis and compromised gas exchange in the corresponding lung segments [1]. 

Some potential causes of unilateral diaphragmatic paralysis include chest trauma, compression, neuropathic processes, inflammatory processes, and iatrogenic or idiopathic causes. The patient has a past medical history of chronic ureteral strictures; therefore, an ileal conduit procedure with urinary diversion was conducted. The patient has a nephrostomy tube that was placed two years ago. An infectious or inflammatory process from this urinary pathology may be related to the patient’s diaphragmatic paralysis or weakness. Numerous systemic diseases can cause inflammation of the phrenic nerve or the diaphragm, resulting in diaphragmatic paralysis. In those with unilateral diaphragmatic paralysis, studies show that there can be an expected 50% decline in forced vital capacity, suggesting susceptibility to further complications [1]. An elevated hemidiaphragm in hospitalized patients, often without symptoms, can arise from lobar collapse, lung surgery, diaphragmatic eventration, or phrenic nerve paralysis linked to various factors such as surgery, tumors, viruses, spine problems, diabetes, autoimmune disorders, and infections - the most common being cardiac surgery. 

Most patients with unilateral diaphragmatic paralysis are asymptomatic. However, patients may present with dyspnea, orthopnea, sleep disturbances, and respiratory failure [4]. The severity of symptoms of hemidiaphragm paralysis or weakness may be influenced by comorbidities such as obesity, pneumonia, chronic obstructive pulmonary disease exacerbations, neuromuscular disorders, and sleep disorders. In obese patients, unilateral diaphragmatic paralysis significantly limits ventilation during exercise, resulting in decreased peak VO_2_, peak work rate, and amount of time able to exercise [5].

Imaging is useful for the diagnosis of unilateral diaphragmatic paralysis. On a chest radiograph, unilateral diaphragm paralysis is evident when one hemidiaphragm is abnormally elevated, with one positioned at or above 2 cm from the other [6]. Although chest radiographs possess a high negative predictive value (93%), their positive predictive value for diagnosis is limited [7]. In cases of uncertainty, the recommended confirmation method is the fluoroscopic "sniff" test, which assesses diaphragmatic movement during forced inspiration. This test is positive in over 90% of patients with unilateral diaphragm paralysis [1]. In this case, the patient demonstrated decreased diaphragmatic excursion and decreased elevation of the left hemidiaphragm during rapid sniff and deep inspiration compared to the right side, confirming the diagnosis of left hemidiaphragm paralysis. A chest tomography (CT scan) can supplement the etiology of a diagnosed unilateral diaphragmatic paralysis by ruling out possible masses and other thoracic etiologies, such as atelectasis [4]. The patient in this case was not found to have any lesions affecting the left hemidiaphragm. Also, the nuclear medicine pulmonary perfusion with ventilation scan showed limited ventilation and perfusion in the left lower lung, which was a result of an elevated left hemidiaphragm. 

For symptomatic patients who present with confounding disease of the lungs, treatment - including non-invasive positive pressure or invasive ventilation - can inadvertently address diaphragm paralysis. It is important to note that this is only a temporary support measure that does not correct or cure the elevated hemidiaphragm [1]. During rapid eye movement (REM) sleep, apneas and hypopnea reduce ventilation, increasing end-tidal CO_2_ concentration. For patients with hemidiaphragmatic paralysis, the ability to ventilate can further worsen during sleep and lead to hypercapnia. Studies show that if patients have increased sleep-disordered breathing, a trial of continuous positive airway pressure (CPAP) or bilevel positive airway pressure (BiPAP) to supplement ventilation could be useful. For the management of unilateral diaphragmatic paralysis in asymptomatic patients, there is no requirement for intervention as long as there are no underlying cardiopulmonary conditions. Instead, periodic clinical observation is recommended for 12 months [1]. In those who have had recent trauma or surgery, watchful waiting can be considered, as the phrenic nerve can be expected to return to baseline after adequate time. It is estimated that the average recovery time for patients who fully recover from diaphragmatic paralysis is over a year, with one study reporting 14.9 ± 6.1 months [8]. It is important to correct the underlying conditions to improve symptoms. However, if a patient persists with symptoms due to hemidiaphragmatic paralysis for more than six months, surgery is an option. The procedure involves suturing the diaphragm into a stable position. Studies have shown that this form of management has proven to improve dyspnea, exercise tolerance, and vital capacity. Studies have also shown positive results with phrenic nerve pacing. The pacemaker stimulates the diaphragm to contract similarly to how it does physiologically, for those without denervation of the diaphragm [1].

## Conclusions

In many instances of unilateral diaphragmatic paralysis, patients may not experience symptoms, and the paralysis is discovered incidentally. This case highlights that unilateral diaphragmatic paralysis is frequently missed in clinical diagnoses due to the lack of symptoms in most patients or the presence of other lung conditions, which can lead to its oversight. Complications may arise if significant co-morbidities are present. Therefore, diagnosing unilateral diaphragmatic paralysis and determining the underlying etiology is important for successful patient outcomes. 
